# *Zingiber officinale* Polysaccharide Silver Nanoparticles: A Study of Its Synthesis, Structure Elucidation, Antibacterial and Immunomodulatory Activities

**DOI:** 10.3390/nano15141064

**Published:** 2025-07-09

**Authors:** Xiaoyu Chang, Huina Xiao, Mingsong Li, Yongshuai Jing, Kaiyan Zheng, Beibei Hu, Yuguang Zheng, Lanfang Wu

**Affiliations:** 1 College of Pharmacology, Hebei University of Chinese Medicine, Shijiazhuang 050200, China; yjs20232108@hebcm.edu.cn (X.C.); yjs20222102@hebcm.edu.cn (H.X.); zhengkaiyan@hebcm.edu.cn (K.Z.); 2 College of Chemical and Pharmaceutical Engineering, Hebei University of Science and Technology, Shijiazhuang 050018, China; mingsongli@hebust.edu.cn (M.L.); jingys@hebust.edu.cn (Y.J.); 3 Traditional Chinese Medicine Processing Technology Innovation Center of Hebei Province, Shijiazhuang 050200, China

**Keywords:** *Zingiber officinale*, nanoparticles, process optimization, characterization, antibacterial activity, immunomodulatory activity

## Abstract

Green-synthesized metal nanoparticles show promise in nanomedicine and material engineering. In this study, the polysaccharide of *Zingiber officinale* (ZOP) was used as a raw material. Through single-factor experiments and a response surface methodology, the optimum synthesis protocol of *Zingiber officinale* polysaccharide silver nanoparticles (ZOP-NPs-AgNPs) was determined as follows: V(AgNO_3_):V(ZOP) = 2.98:1, 59.79 °C, 3 h, pH 9, and 20 mL NaCl, achieving a 92.51% silver chelation rate. Structure analysis revealed that ZOP-NPs-AgNPs were spherical or quasi-spherical, with a particle size < 20 nm and a face-centered cubic crystal structure, which has good thermal stability. Subsequent studies explored the antibacterial and immunomodulatory effects of ZOP-NPs-AgNPs. The minimum inhibitory concentration (MIC) of ZOP-NPs-AgNPs against *Escherichia coli* and *Staphylococcus aureus* was determined to be 0.5000 mg/mL and 0.0310 mg/mL, respectively, while the minimum bactericidal concentration (MBC) was 0.5000 mg/mL and 0.0310 mg/mL, respectively. Additionally, ZOP-NPs-AgNPs significantly enhance RAW264.7 cell proliferation and phagocytosis and boost IL−1β, IL−6, NO, and TNF-α production. This confirms that ZOP can act as a green reductant and stabilizer, offering a new method for green nano-silver synthesis. This provides a sustainable way to produce antibacterial products and functional foods, and offers useful references for eco-friendly nano-silver applications.

## 1. Introduction

In recent years, nanomaterials have been widely studied, among which metal nanomaterials are an important research focus. They generally refer to materials with particle sizes of 1–100 nm, which have a unique quantum-size effects and surface effects, unlike ordinary metallic substances. Silver nanoparticles (AgNPs) have unique physical and chemical properties and are widely used in medicine, furnishing materials, and other fields [1]. Because of their smaller size and larger specific surface area, they have greater advantages in the antibacterial activity. In recent years, the progression of nanotechnology has propelled the extensive application of metal nanoparticles across various domains, such as biomedicine, food packaging, and catalysis [2,3,4,5]. AgNPs, in particular, have garnered significant attention due to their potent bactericidal properties against a wide spectrum of bacteria, including both drug-resistant and drug-sensitive strains (Gram-positive and Gram-negative bacteria), positioning them as promising candidates for use in pharmaceuticals and food packaging. At present, common methods for synthesizing AgNPs encompass physical processes, chemical reduction techniques, and biological reduction approaches [6]. Physical methods typically necessitate high-performance equipment, operate under stringent conditions, and incur high costs, which poses challenges for large-scale production. Meanwhile, conventional chemical reduction techniques often employ potent reducing agents such as sodium borohydride (NaBH_4_) [7] or trisodium citrate [8] under rigorously controlled conditions (e.g., temperature, pH) and have been broadly applied in the synthesis of AgNPs with specific sizes and morphologies. Notwithstanding, these conventional methods frequently involve toxic chemicals, demand strict reaction control, and may leave behind harmful residues [9]. ISO international standards highlight that employing fewer reagents and more eco-friendly techniques is the optimal choice [10]. Consequently, it is of vital importance to develop a method for synthesizing AgNPs that possess low toxicity and can slowly release Ag^+^. Adhering to the principles of “green chemistry”, which advocates for the preferential use of natural and nontoxic components, represents an effective strategy to achieve this objective [11].

The biological reduction method has emerged as a promising approach. In this method, simple, economical, and eco-friendly natural materials serve as reducing agents for synthesizing AgNPs. These natural materials also act as stabilizing agents, preventing excessive aggregation of AgNPs, thereby enhancing their biocompatibility [12,13,14]. Currently, various biomacromolecules have been employed to stabilize dispersed AgNPs, including pectin (Akebia trifoliata var.) [15], chitosan [16], cellulose [17], fungal polysaccharides [18], and carboxymethyl cellulose [19], among others. Polysaccharides, owing to their wide availability and excellent biocompatibility, have become ideal natural reducing agents. AgNPs prepared using polysaccharides as both reducing and stabilizing agents not only possess inherent advantages but may also exhibit additional biological activities [20].

*Z. officinale* is a perennial herb of Zingiberaceae, which contains many active components, such as polysaccharides, polyphenols, terpenoids, gingerol, and its derivatives. It can effectively fight against many pharmacological effects, such as cardiovascular diseases, ulcers, tumors, indigestion, and inflammation [21,22]. Polysaccharides are one of the main functional active components in ginger, which has a significant developmental value and application prospect in medicine, hygiene, food, condiments, and cosmetics [23]. Numerous data show that ZOP has many biological activities, such as antitumor, antioxidant, and antiglycemic, and has the advantages of high safety and fewer toxic and side effects [24,25].

In response to the rising demand for sustainable and eco-friendly practices in nanotechnology development and considering the unique properties of ZOP and silver nanoparticles, this study employs the natural product ZOP as a reducing agent for silver nanoparticle synthesis. In preliminary studies, Z. officinale polysaccharides were extracted, and using ZOP and ZOP-NPs as reducing and stabilizing agents, ZOP-AgNPs and ZOP-NPs-AgNPs were synthesized. The physicochemical properties of ZOP, ZOP-AgNPs (0.5%, *w*/*v*; 1%, *w*/*v*), and ZOP-NPs-AgNPs (0.5%, *w*/*v*; 1%, *w*/*v*) were comprehensively analyzed using UV-visible spectrophotometer (UV-Vis), Fourier transform infrared spectroscopy (FT-IR), scanning electron microscopy (SEM), transmission electron microscopy (TEM), and X-ray diffraction (XRD). Comparative studies on antioxidant and antibacterial activities revealed that ZOP-NPs-AgNPs exhibited superior performance to ZOP, ZOP-NPs, and ZOP-AgNPs [26]. To further explore the properties and potential applications of ZOP-NPs-AgNPs, through optimization using single-factor experiments and the response surface methodology (RSM), we obtained a single target sample of ZOP-NPs-AgNPs synthesized under specific optimal parameters (an AgNO_3_-to-polysaccharide volume ratio of 3:1, temperature of 60 °C, and time of 3 h). Based on the optimized conditions, the structure of ZOP-NPs-AgNPs was further analyzed, and its antibacterial and immunomodulatory activities were systematically evaluated. This study aims to meet the urgent need for the development of environmentally friendly and efficient alternative synthetic methods. By significantly enhancing the antibacterial and immunomodulatory properties of silver nanoparticles, it holds great potential to drive the future development of medical treatment, food preservation, and environmental protection. This study not only comprehensively and deeply analyzes the characteristics and application prospects of ZOP-NPs-AgNPs but also develops a green synthesis strategy, laying a solid theoretical foundation for the wide application of silver nanoparticles in various industries.

## 2. Materials and Methods

### 2.1. Materials and Reagents

*Z. officinale* (No. 2019031401) was purchased from AnguoYaoyuan Trading Co., Ltd. (Hebei, China). The sample was identified as the rhizome of *Zingiber officinale* Roscoe by professor Yuguang Zheng (Department of Pharmacy, Hebei University of Chinese Medicine). A voucher specimen was deposited in the College of Chemistry and Pharmaceutical Engineering, Hebei University of Science and Technology, China. AgNO_3_ was purchased from Shanghai Aladdin Biochemical Technology Co., Ltd. (Shanghai, China), tryptone and agar powder were purchased from Beijing Auboxing Biotechnology Co., Ltd. (Beijing, China), *Escherichia coli* and *Staphylococcus aureus* were obtained from Beijing Biological Preservation Center (Beijing, China)., and RAW264.7 cells were obtained from the Cell Bank of the Chinese Academy of Sciences (Shanghai, China). All other chemicals and reagents were analytical grade.

### 2.2. Preparation of ZOP

The ZOP samples used in this experiment were derived from the previously conducted experiments [26], and the initial structural characterization of ZOP was carried out.

### 2.3. Preparation of Z. officinale Polysaccharide Nanoparticles (ZOP-NPs)

In this experiment, ZOP-NPs were first prepared by nano-precipitation method, and then ZOP-NPs were used to synthesize AgNPs. A total of 1.5 g ZOP was dissolved in 100 mL distilled water to obtain 1.5% (*w*/*v*) polysaccharide solution. Under magnetic stirring, the polysaccharide solution was dropped dropwise into 10 times the volume of absolute ethanol, with stirring continuing for 2 h, and the solution was concentrated and dried to obtain ZOP-NPs [27].

### 2.4. Preparation of ZOP-NPs-AgNPs

#### 2.4.1. Single-Factor Experiment

AgNO_3_ solution (0.05 mol/L), ZOP-NPs solution (1 mg/mL), and NaCl solution (1 mg/mL) were prepared. The polysaccharide solution, AgNO_3_ solution, and NaCl solution were measured in proportion, and pH was adjusted with ammonia water; the final solution was irradiated under 365 nm violet light and stirred for a certain period of time to obtain ZOP-NPs-AgNPs [28]. In this experiment, silver chelation rate was used as the index, and five factors were considered, namely, the volume ratio of AgNO_3_ solution and ZOP-NPs solution (1.5:1, 2:1, 2.5:1, 3:1, 3.5:1), the volume of NaCl (5, 10, 15, 20, 25/mL), temperature (20, 40, 60, 80, 100/°C), time (1, 2, 3, 4, 5/h), and pH value (7, 8, 9, 10, 11). While optimizing each factor, other factors remained stable. Silver ion chelation rate was determined by Volhard method. Reaction of [NH_4_ Fe(SO_4_)●12 H_2_O] is:

(1) $${\mathrm{Ag}}^{+}+{\mathrm{SCN}}^{-}\to \mathrm{Ag}\mathrm{SCN}$$

(2) $${\mathrm{Fe}}^{3+}+{\mathrm{SCN}}^{-}\to \mathrm{Fe}{\mathrm{SCN}}^{2+}$$

A total of 20 mL of ZOP-NPs-AgNPs solution was measured, and four times the volume of anhydrous ethanol was added to the solution. Then, the solution was allowed to stand for 20 min before being centrifuged at 8000 r/min for 10 min. After that, 20 mL of supernatant was transferred to the solution, an appropriate amount of ammonium thiocyanate standard solution (0.76 mg/mL) was added, the titration was performed, the color of the solution and the volume of the standard solution consumed were recorded, and Equation (1) was used to calculate the chelation rate of silver ions.

(3) $$\;A\left(\%\right)=(C_{0}\;-\;C/C_{0})\;\times \;100\%$$

A represents the chelation rate (%), C_0_ is the concentration of silver ion in the solution before the reaction (mg/mL), and C is the concentration of silver ion in the solution after the reaction (mg/mL).

#### 2.4.2. Box–Behnken Response Surface Methodology

Based on the results of the single-factor experiment, the Box–Behnken test factor level table was designed (Table 1). In this study, the silver chelation rate was used as the response value, 17 groups of experiments were analyzed, and a response surface model was established to obtain the best preparation process.

### 2.5. Structural Characterization of ZOP-NPs-AgNPs

#### 2.5.1. UV-Vis Spectroscopy Analysis

During the preparation of AgNPs, 5 mL of ZOP-NPs-AgNPs solution was measured every 1 h. Scanning and recording spectra were in the range of 200–800 nm by a UV-vis spectrophotometer [29].

#### 2.5.2. FT-IR Spectroscopy Analysis

ZOP-NPs-AgNPs sample powder and KBr were dried at 60 °C to constant weight. The sample was made into transparent and uniform tabletting by the KBr tabletting method, and FT-IR spectra were analyzed by an S−100 spectrometer (PerkinElmer, Waltham, MA, USA) in the range of 4000–400 cm^−1^.

#### 2.5.3. SEM Analysis

ZOP-NPs-AgNPs were deposited on a sample table and then analyzed by a field emission scanning electron microscope (JSM-7610 F) under 3.0 kV accelerated voltage and high vacuum.

#### 2.5.4. TEM Analysis

ZOP-NPs-AgNPs were observed at 100 keV using a transmission electron microscope analyzer (JEM-2100, JEOL, Tokyo, Japan).

#### 2.5.5. XRD Analysis

The crystal structure of the samples was analyzed by an X-ray diffractometer model (XRD-6000, Shimadzu, Kyoto, Japan). Cu-Kα ray (λ = 0.15406 nm) was used as the target, the tube voltage was 40 kV, the tube current was 30 mA, the scanning rate was 5 °C/min, and the diffraction Angle was 2θ = 10–90° [30].

#### 2.5.6. TGA Analysis

TGA-DSC (TA Instruments Ltd., Q600, New Castle, DE, USA) was used for thermogravimetric analysis. The temperature was increased from room temperature to 800 °C, and the heating rate was 10 °C/min.

### 2.6. Antibacterial Activity of ZOP-NPs-AgNPs

#### 2.6.1. The Determination of the Diameter of the Bacteriostatic Zone

The antibacterial activity of ZOP-NPs-AgNPs was evaluated using the agar well diffusion method. In both antibacterial tests (*Escherichia coli* and *Staphylococcus aureus*), 20 mg/mL and 40 mg/mL of ZOP-NPs-AgNPs were prepared, respectively. The control groups included AgNO_3_ gel, ampicillin, 20 mg/mL, and 40 mg/mL of AgNO_3_. In the experimental procedure, after the agar in the solid culture medium had coagulated, the diluted bacterial suspension was evenly spread on the surface of the solid culture medium by streaking in a “Z” pattern with cotton swabs (three times). Subsequently, the Oxford cup was placed vertically on the solid culture medium, and 100 μL of each sample solution was dispensed into the cup. The samples were then incubated at 37 °C for 24 h in a constant temperature incubator. Eventually, the antibacterial activity of ZOP-NPs-AgNPs was evaluated and quantified by the diameter (cm) of the inhibition zones [31].

#### 2.6.2. The Minimum Inhibitory Concentration (MIC) of ZOP-NPs-AgNPs

The MIC values, which represent the lowest extract concentrations that prevent the visible growth of microorganisms, were determined by a twofold microdilution test in combination with the colorimetric Resazurin assay. Resazurin solution, which is blue, is commonly used as an acid-base indicator (pH 3.8 (orange) − 6.5 (deep purple)) and a redox indicator. Resazurin solution can penetrate cells, and it will be irreversibly reduced to pink in the environment where bacteria grow. The concentration of the solution before it turns pink is the MIC.

Using Resazurin solution as an indicator, AgNO_3_ (1 mg/mL) and ampicillin (1 mg/mL) served as positive controls, while DMSO and normal saline were used as negative controls. In a 96-well plate, a twofold continuous diluted sample solution (1–1/8192 mg/mL) was prepared and dispensed into each well. Subsequently, Resazurin indicator (0.07 g/mL) and bacteria solution were added to each well. After thoroughly mixing the contents of each well, the 96-well plate was covered with a sterile plate and incubated at 37 °C in a constant temperature incubator. The color of each well in the 96-well plate was observed every 12 h. If the color in the hole turned red or pink, it indicated that bacteria grew in the well. If the color in the hole was still blue, it indicated aseptic growth, and the concentration at which the first well turned pink was considered the MIC [32].

#### 2.6.3. The Minimum Bactericidal Concentration (MBC) of ZOP-NPs-AgNPs

Based on the aforementioned experimental results, the sample concentration corresponding to blue wells in the 96-well plate was selected for the follow-up experiment. The ZOP-NPs-AgNPs with corresponding concentrations were mixed with bacterial liquid and sterile water, and then evenly smeared on a solid culture medium. After incubation at 37 °C for 24 h in a constant temperature incubator, the bacterial growth was observed. If the number of bacterial colonies in the plate was less than 5, the corresponding sample concentration was recorded as the effective bactericidal concentration, and the effective minimum bactericidal concentration of each strain was the MBC.

### 2.7. Immunomodulatory Activity of ZOP-NPs-AgNPs

#### 2.7.1. Culture of RAW264.7 Cells

RAW264.7 cells were obtained from Shanghai Cell Bank of Chinese Academy of Sciences and cultured in DMEM medium (basic medium: fetal bovine serum: penicillin-streptomycin solution = 90:10:1) at 37 °C and 5% CO_2_. After 24 h, the fresh culture medium was changed, and it continued to be cultured. When the cells grew to 80–90% of the bottom area of the culture bottle and the cells were in good shape, they were subcultured.

#### 2.7.2. Cell Proliferation Experiment

The effect of ZOP-NPs-AgNPs on macrophage viability was detected by the CCK-8 method. After adjusting the concentration to 2 × 10^5^ cells/mL, the cells were seeded into 96-well plates with 100 μL per well and cultured in a cell incubator. After 24 h, 100 μL of ZOP-NPs-AgNPs (1–500 μg/mL) with different concentrations was used to replace the culture medium, and lipopolysaccharide (LPS) (1 μg/mL) was added to the positive control group. After 20 h, 0.5 mg/mL CCK-8 solution was added to the 96-well plate. After continuing culture for 4 h, the supernatant was removed, 150 μL of DMSO was added to each well, and the plate was shaken to fully dissolve the purple formazan crystals. The absorbance value of each well was detected by a microplate reader at 490 nm [33].

#### 2.7.3. Cell Phagocytosis Experiment

RAW264.7 cells in the logarithmic growth phase were trypsinized and diluted to a concentration of 5 × 10^5^ cells/mL DMEM culture medium. A 150 μL suspension was placed in a 96-well plate and cultured for 24 h. Subsequently, 50 μL of ZOP-NPs-AgNPs solution (1–500 μg/mL) at varying concentrations was added to each well, followed by continued incubation for an additional 24 h. Then the culture medium was removed, and the cells were washed three times with PBS (0.01 M, pH 7.4). Then, 100 μL of cell lysis buffer was added and incubated overnight at room temperature. The absorbance was measured at 540 nm by a microplate reader. The blank control group contained only DMEM medium, while the positive control group was LPS medium [34].

#### 2.7.4. The Effect Test of NO Secretion by Macrophages

To determine the NO production of RAW264.7 cells, RAW264.7 cells in logarithmic phase were digested with trypsin and placed in a 96-well plate. A total of 50 μL ZOP-NPs-AgNPs solution (1–500 μg/mL) was added to the culture for 24 h; then, 100 μL of the culture solution was transferred to a new plate, the Griess reagent was added to the new plate, and the absorbance was measured at 540 nm. The blank control group contained only DMEM medium, while the positive control group was LPS. The NO production was calculated according to the standard curve made with NaNO_2_ as the standard [35].

#### 2.7.5. Assay of Cytokines

RAW264.7 cells were treated with varying concentrations of ZOP-NPs-AgNPs for 24 h, following the method described in the cell proliferation experiment (Section 2.7.2). The secretion of TNF-α, IL-1β, and IL-6 cytokines in the cell culture medium was detected according to the instructions of the ELISA kit (from ABclonal, Wuhan, China).

#### 2.7.6. Data and Analysis

Excel was employed for the initial organization and sorting of the test data. Design Expert 8.0 was used to complete the response surface test design and correlation analysis. SPSS 16.0 was used to analyze the significance, and Origin 8 was used to draw the data. The level of statistical significance was set at *p* < 0.05.

## 3. Results and Discussion

### 3.1. Preparation of ZOP-NPs-AgNPs

#### 3.1.1. Single-Factor Experiment Analysis

**Figure 1:**
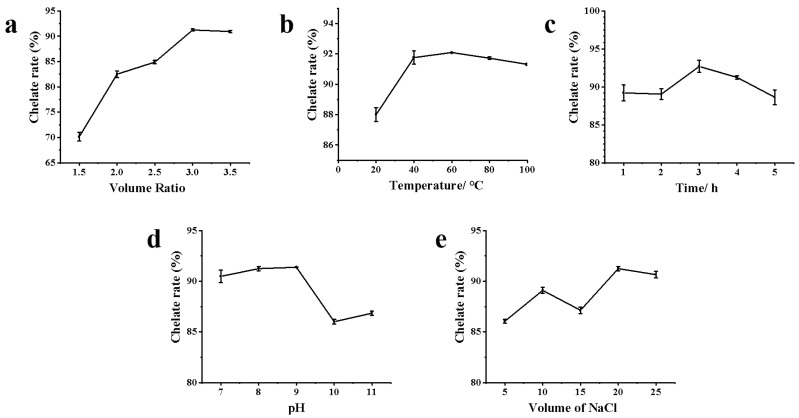
The chelation rate of ZOP-NPs-AgNPs under different influencing factors: (**a**) the volume ratio of AgNO_3_ and polysaccharide; (**b**) temperature; (**c**) time; (**d**) pH; (**e**) the volume of NaCl.

The chelation rate increased with the increase in the volume ratio of AgNO_3_ and ZOP-NPs solution, reaching its maximum when the volume ratio is 3:1 (Figure 1a). Before and after that, there was a downward trend. Studies have shown that as the concentration of AgNO_3_ increases, more silver nanoparticles are formed in the solution, resulting in a broader ultraviolet absorption peak [36]. However, with the increase in the volume ratio, the chelation rate eventually tends to become flat or even decrease, which may be due to the maximum chelation between polysaccharide and Ag^+^ in the solution or to the transformation of silver nanoparticles into silver oxide nanoparticles under the condition of insufficient stabilizing reagents [37,38]. Therefore, the volume ratio of AgNO_3_ and polysaccharide solution was selected as 3:1 for further research.

The chelation rate increased with the rise in water bath temperature, peaking at 60 °C (Figure 1b). However, further temperature increases led to a decline in the chelation rate, likely due to excessive aggregation of ZOP-NPs-AgNPs, which interfered with chelation rate determination. Therefore, an extraction temperature from 50 to 70 °C was determined to be suitable for further experiments.

As shown in Figure 1c, the chelation rate increased with the increase in ultraviolet irradiation time. At the same time, the solution changed its color to dark brown or black, which reached the highest rate when the ultraviolet irradiation time was 3 h; however, the chelation rate decreased with the increase in ultraviolet irradiation time, which may be due to the agglomeration of ZOP-NPs-AgNPs with the increase in time, which affected the determination of the chelation rate.

Under alkaline conditions, Ag^+^ reacts with -OH to form Ag_2_O. The -COOH and -OH groups lose H^+^ to form salts, which then form complexes with Ag_2_O. The -CHO and -C=O groups in ZOP reduce Ag_2_O to AgNPs by reacting with the Ag_2_O complex. In this process, -CHO is the primary reducing agent, and the original -COOH, -OH, and the generated-COOH play a stable role in the formation of AgNPs [39]. Analysis of Figure 1d shows that the chelation rate increased with the solution pH. A significant pH increase facilitated AgNPs formation, likely due to a larger number of available functional groups in the polysaccharide solution for binding, thus promoting AgNPs formation [40]. However, at pH 10 and 11, the chelation rate decreased, possibly because the excessive alkalinity from added ammonia water disrupted the polysaccharide structure, affecting AgNPs formation and, consequently, the chelation rate.

Based on Figure 1e, when the volume of NaCl added was 20 mL, the chelation rate was the highest, but as the volume of NaCl increased, the chelation rate decreased. It may be that the concentration of Cl^−^ in the solution increased, which affected the generation of AgNPs, resulting in chelation rate decrease.

#### 3.1.2. Response Surface Results and Contour Plots

From the single-factor experiment results for the above five groups, the volume ratio of AgNO_3_ to polysaccharide solution, water bath temperature, and ultraviolet time have a great influence on the preparation process of ZOP-NPs-AgNPs, while the pH value and NaCl volume can be fixed at 9 and 20 mL. According to the principle of three factors and three levels, the volume ratios of AgNO_3_ and polysaccharide solution were determined as 2.5:1, 3:1, and 3.5:1; the experimental temperatures were 50, 60, 70 °C; The experimental times were 2, 3, 4 h. A model was established with silver chelation rate as the response value, and 17 groups of experiments were conducted (Table 2).

The Box–Behnken experimental design method was used to fit the relationship between each factor and response value by using the equation, and the equation was analyzed to explore the best process parameters. The fitting regression equation was obtained:

Y = 92.51 − 0.26 A − 0.76 B + 0.0075 C − 0.41 AB + 0.47 AC − 0.39 BC − 2.63 A^2^ − 1.3 B^2^ − 0.11 C^2^ (4)

The *p*-values were used as a tool to check the significance of each coefficient. From the experimental results (Table 3), the model’s *p*-value was less than 0.0001, indicating a significant model fit. The lack-of-fit value was 0.6649, indicating that it was insignificant relative to the pure error (*p* > 0.05). The *p*-values of A, AB, AC, BC, A^2^, B^2^, and C^2^ were below 0.05, indicating these factors significantly affected the chelation rate. According to the F-value, the volume ratio of AgNO_3_ to polysaccharide solution had the most significant impact on ZOP-NPs-AgNPs preparation, followed by water bath temperature and then violet light irradiation time. The fitting coefficient was R^2^ = 0.9879, which suggested that the quadratic model was significant. According to the response surface methodology, the optimum preparation technology of ZOP-NPs-AgNPs was as follows: the volume ratio of AgNO_3_ to polysaccharide was 2.98:1, the water bath temperature was 59.79 °C, and the irradiation time of purple light was 3 h.

**Figure 2:**
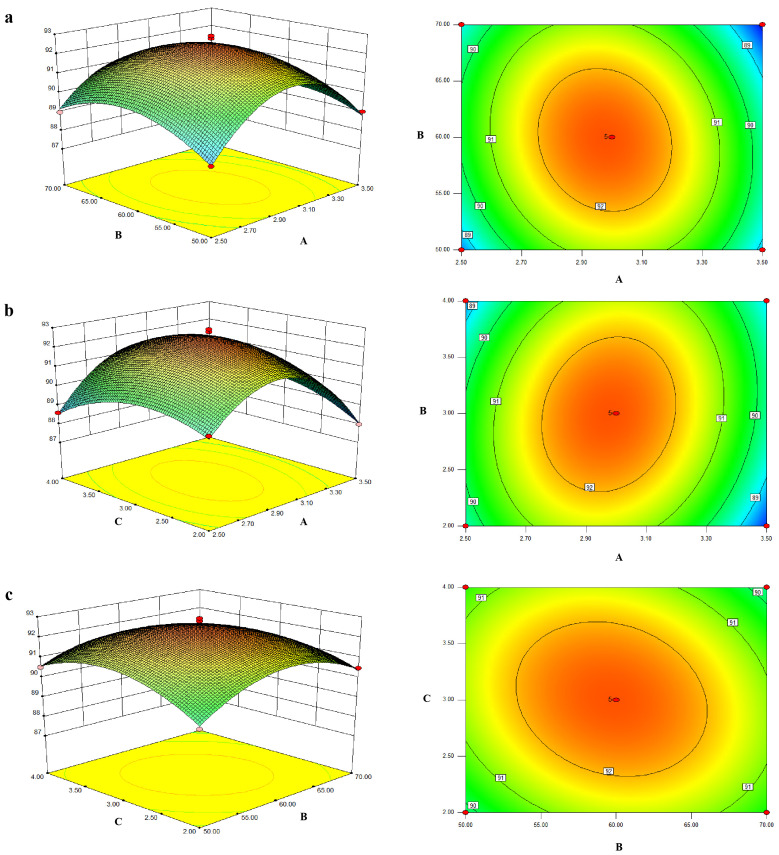
(**a**) The effect of the interaction between AgNO_3_ and the polysaccharide solution volume ratio and temperature on the chelation rate. (**b**) The effect of AgNO_3_ and polysaccharide solution volume ratio and time interaction on the chelation rate. (**c**) The effect of the interaction between temperature and time on the chelation rate.

Through the three-dimensional response surface and contour map of model fitting, the relationship between different influencing factors and silver chelation rate was visualized (Figure 2). The response surface diagram illustrates the combined effects of two continuous variables on the silver chelation of ZOP-NPs-AgNPs, with the third variable held constant at zero. The results (Figure 2a) show the effects of the AgNO_3_ and polysaccharide solution volume ratio and temperature on chelation rate and their interactions. As the extraction temperature and volume ratio rose, the chelation rate of ZOP-NPs-AgNPs increased in a parabolic direction, indicating that the model’s maximum value was at the center.

The contour plot also reveals a significant interaction between the volume ratio of AgNO_3_ and polysaccharide solution and time. In addition, the chelation rate increases with the increase in the volume ratio, but with the extension of time, the trend of the chelation rate increasing is not obvious (Figure 2b). From the response surface and contour plots of temperature and time (Figure 2c), it is evident that the chelation rate increased with higher temperatures and longer times. Overall, the order of importance for the three factors in AgNP synthesis was as follows: Volume ratio (AgNO_3_: polysaccharide) > Temperature > Irradiation Time.

To verify the model and assess the accuracy of the optimization results, experiments were conducted under the model-predicted conditions: an AgNO_3_-to-polysaccharide volume ratio of 3:1, temperature of 60 °C, and time of 3 h. The resulting chelation rate of 92.17% was very close to the predicted value of 92.51%, confirming the model’s validity.

### 3.2. Structural Characterization of ZOP-NPs-AgNPs

#### 3.2.1. The Determination of the Particle Size and Silver Chelating Rate

Polysaccharides can be used not only as a reducing agent to reduce silver ions but also as a stabilizer to prevent the agglomeration of AgNPs during the formation of AgNPs [41]. The silver chelation rate of the polysaccharide was 92.17%, with a particle size below 20 nm, a PDI of 0.299, and a zeta potential of −32.83 mV. The silver chelation rate of ZOP-NPs-AgNPs prepared under the optimum conditions is higher than that of ZOP-NPs-AgNPs prepared before (68.70–82.12%) [26].

#### 3.2.2. UV-Vis Analysis

**Figure 3:**
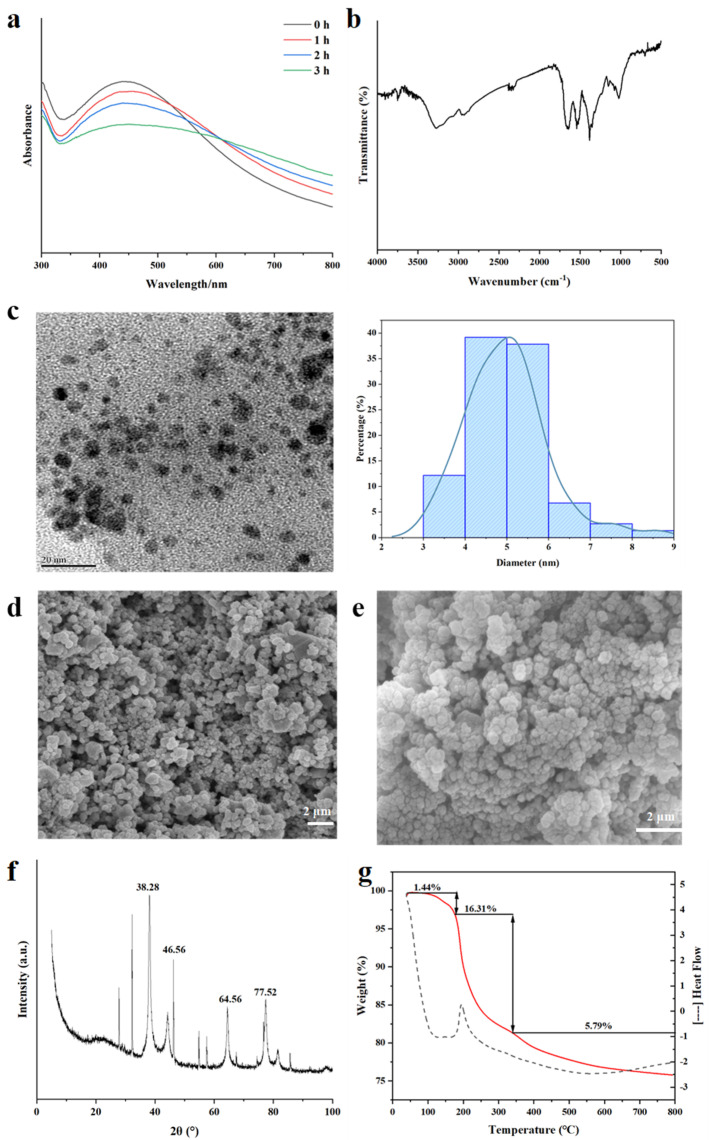
Structural characterization of ZOP−NPs−AgNPs. (**a**) UV−vis. (**b**) FT−IR. (**c**) TEM and size statistics. (**d**,**e**) SEM. (**f**) XRD. (**g**) TGA (the red line represents weights and the dots represents heat flow).

AgNPs have characteristic absorption peaks in the wavelength range of 300–800 nm [42]. The UV-Vis spectra (Figure 3a) show that there was an obvious absorption peak at about 430 nm, which was the unique surface plasmon resonance of AgNPs [43], confirming the formation of spherical AgNPs in the solution. The intensity of the absorption peak increased gradually with the increase in time, indicating the increase in AgNPs in the solution [44]. The single broad peak in the spectrum suggests the polydispersion of ZOP-NPs-AgNPs. At the same time, we found that some researchers experienced a similar phenomenon in the study of synthesizing silver nanoparticles from *Rosa roxburghii* Tratt fruit polysaccharide [45].

#### 3.2.3. FT-IR Analysis

The FT-IR spectrum of ZOP-NPs-AgNPs (Figure 3b) exhibits distinct alterations in characteristic absorption peaks compared to pure ZOP [26], confirming structural changes upon AgNPs formation. Key observations include the following: narrowing of the -OH stretching vibration at 3282 cm^−1^, suggesting hydroxyl group involvement in AgNPs reduction and stabilization; significant attenuation of the -CH absorption peak at 2943 cm^−1^, indicating strong interactions between AgNPs and aliphatic chains [41]; and intensified -COO^−^ vibration at 1630 cm^−1^, coupled with weakened peaks at 1642.09 cm^−1^ (C=O), 1389 cm^−1^ (C-H), and 1017 cm^−1^ (C-O), collectively demonstrating the participation of surface hydroxyl and carboxyl groups in AgNPs synthesis. This trend aligns with reported mechanisms for polysaccharide-stabilized AgNPs [40,46], notably matching spectral shifts observed by Ma et al. [47] in soluble soybean polysaccharide-templated AgNPs.

#### 3.2.4. TEM Analysis

TEM analysis of ZOP-NPs-AgNPs revealed that the solution primarily contained spherical AgNPs (Figure 3c). The dispersion of ZOP-NPs-AgNPs was good, the particle size of AgNPs was less than 20 nm, and the signs of aggregation were not obvious. Moreover, the mean diameter and standard deviation of ZOP-NPs-AgNPs were 4.99 ± 0.99 nm. The results indicated that ZOP-NPs function as a stabilizer by encapsulating the surface of nanoparticles, thereby reducing the surface activity of AgNPs and preventing their aggregation [48]. The experimental results showed that the AgNPs prepared under optimal conditions have advantages such as high silver chelation rates, small particle size, and good dispersion. Higher silver chelation rates and smaller particle sizes translate to larger specific surface areas, enabling AgNPs to have a larger contact area and, thus, exhibit better biological activity.

#### 3.2.5. SEM Analysis

SEM of ZOP-NPs-AgNPs (Figure 3d,e) shows that the shape of AgNPs is very close to spherical, but there were also a small number of block structures. The presence of these block-like structures may be attributed to the aggregation of AgNPs during long-term storage or the freeze-drying process.

#### 3.2.6. XRD Analysis

The crystallinity of formulated ZOP-NPs-AgNPs was tested by XRD (Figure 3f)). ZOP-NPs-AgNPs showed four prominent diffraction peaks, which were located at (2θ) 38.28° [49], 47.56° [42], 64.56° [50], and 77.52° [51], respectively, corresponding to the four planes of (111), (200), (220), and (311) of face-centered cube (FCC) [42]. The sharp and narrow peaks indicated the crystalline nature of ZOP-NPs-AgNPs, confirming the successful synthesis of AgNPs. These characteristic peaks have been widely reported by researchers and were thought to be related to the crystallization of organic phases on the surface of AgNPs [52,53]. Additionally, some minor peaks were observed in Figure 3f, which may be due to improper handling or the introduction of impurities during the synthesis process or, possibly, due to the failure to remove lipids and proteins during polysaccharide extraction [54].

#### 3.2.7. TGA Analysis

The thermal stability of the prepared ZOP-NPs-AgNPs was investigated using thermogravimetric analysis (TGA). The process can be divided into three distinct stages (Figure 3g). The initial degradation stage of ZOP-NPs-AgNPs occurred at about 38−165 °C, in which water was mainly lost, and the weight loss rate was 1.44%. The second stage mainly occurred at about 165−330 °C, which was mainly caused by the decomposition of the polysaccharide itself, and the weight loss rate was 16.31%. The weight loss rate in the third stage was 5.79%. These observations indicate that the mass loss of AgNPs was primarily due to the volatilization of water and the degradation of the polysaccharide. This also confirms that the polysaccharide effectively encapsulated the AgNPs, which is consistent with previous results. During the synthesis of AgNPs, polysaccharide nanoparticles played a protective and stabilizing role, encapsulating AgNPs and preventing aggregation [55].

### 3.3. Antibacterial Activity of ZOP-NPs-AgNPs

#### 3.3.1. Determination of Diameter of Bacteriostatic Zone

**Figure 4:**
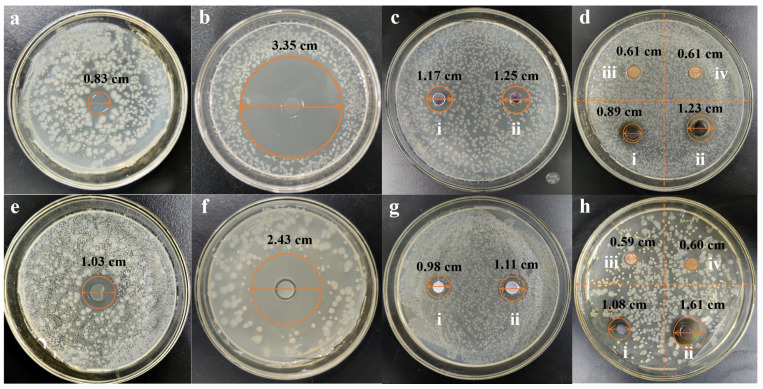
*Escherichia coli* (**a**–**d**) and *Staphylococcus aureus* (**e**,**f**) inhibition zone diameter. (**a**,**e**) AgNO_3_ gel. (**b**,**f**) Ampicillin. (**c**,**g**) AgNO_3,_ (i) 20 mg/mL AgNO_3_, and (ii) 40 mg/mL AgNO_3_. (**d**,**h**) ZOP and ZOP-NPs-AgNPs, (i) 20 mg/mL ZOP-NPs-AgNPs, (ii) 40 mg/mL ZOP-NPs-AgNPs, (iii) 20 mg/mL ZOP, and (iv) 40 mg/mL ZOP.

The antibacterial activity of ZOP-NPs-AgNPs was investigated. The strains *Escherichia coli* and *Staphylococcus aureus* were selected in this experiment (Figure 4). The results indicated that ZOP did not show antibacterial activity. The inhibition zones of ZOP-NPs-AgNPs against *Escherichia coli* (Figure 4d) and *Staphylococcus aureus* (Figure 4h) were 0.89 cm (20 mg/mL), 1.23 cm (40 mg/mL), 1.08 cm (20 mg/mL), and 1.61 cm (40 mg/mL), respectively. According to the results of *Staphylococcus aureus*, ZOP-NPs-AgNPs had better antimicrobial activity than AgNO_3_. This may be due to the higher content of silver in the prepared AgNPs, and because they were nanoparticles with a larger specific surface area, they can better contact the cell membrane and cell wall, destroy their structure and permeability, and at the same time, they can penetrate bacteria, attack the respiratory chain, hinder cell metabolism and cell division, and promote cell apoptosis [56]. From the determination of the inhibition zone diameter of *Escherichia coli* and *Staphylococcus aureus*, it can be seen that ZOP-NPs-AgNPs had a better inhibition effect on *Escherichia coli*. According to the research under different concentrations, the greater the concentration of ZOP-NPs-AgNPs, the larger the inhibition area diameter, indicating that it has a stronger inhibition effect on the growth of bacteria. Affes et al. and He et al. also observed marked dose-dependent antibacterial activities of AgNPs against Gram-positive and Gram-negative bacteria [57,58].

#### 3.3.2. The MIC of ZOP-NPs-AgNPs

**Figure 5:**
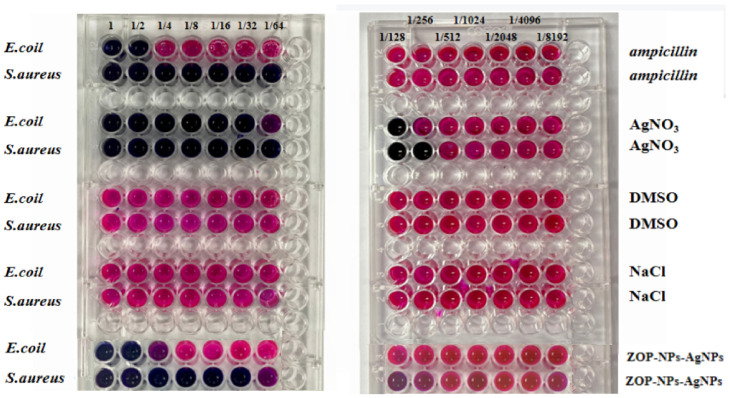
The MIC of ZOP-NPs-AgNPs (*E. coli*: *Escherichia coli*, *S. aureus*: *Staphylococcus aureus*). Initial concentration is 1 mg/mL.

In this experiment, Resazurin was used as an indicator to determine the MIC of ZOP-NPs-AgNPs, and two positive controls and two negative controls were set. The positive controls were ampicillin and AgNO_3_, and the negative controls were DMSO and NaCl. According to the experimental results (Figure 5), DMSO and NaCl had no inhibitory effect on either of the bacteria, and the MIC of ZOP-NPs-AgNPs against *Escherichia coli* and *Staphylococcus aureus* was 0.5000 mg/mL and 0.0310 mg/mL, respectively. The results showed that ZOP-NPs-AgNPs had a better antibacterial effect on *Staphylococcus aureus*. The reason for this phenomenon may be related to the differences in strains, which is consistent with the findings of Liu et al. [59]. *Staphylococcus aureus* is Gram-positive bacteria and *Escherichia coli* is a Gram-positive bacterium, and Escherichia coli is a Gram-negative bacterium. There were differences in AgNPs susceptibility between the two types of bacteria, which may be related to their cellular surface properties and interactions with AgNPs. Chung et al. [60] observed that the negative charge on the cell surface of Gram-negative bacteria was higher than that of Gram-positive bacteria. Therefore, the interaction between Gram-positive bacteria and silver nanoparticles is obviously stronger than that with Gram-negative bacteria, and these conclusions were also consistent with these experimental results. In addition, Gram-negative bacteria have an outer membrane made of lipid, protein, and LPS that serves as a barrier and effectively shields antibacterial medications. However, Gram-positive bacteria do not have an outer membrane as part of their cell wall [61,62].

#### 3.3.3. The MBC of ZOP-NPs-AgNPs

The MBC of ZOP-NPs-AgNPs against *Escherichia coli* and *Staphylococcus aureus* was determined according to MIC results. The lowest MBC of ZOP-NPs-AgNPs to the tested strain was 0.0310 mg/mL to *Staphylococcus aureus*, and no colonies grew in solid medium at this concentration, which indicated that ZOP-NPs-AgNPs had the strongest bactericidal effect on this strain. The MBC (0.5000 mg/mL) for *Escherichia coli* was higher than that for *Staphylococcus aureus*, and the experimental results were consistent with the MIC values.

To evaluate the antibacterial effect of silver nanoparticles synthesized from natural polysaccharides, El-Naggar et al. [63] produced silver nanoparticles by reducing AgNPs with *Chlorella vulgaris* soluble polysaccharides and found that the synthesized AgNPs solutions had an inhibitory effect on *Bacillus, Erwinia, and Candida.* It may be that AgNPs affect antibacterial potentiality by penetrating the cell wall of bacteria and causing damage through interactions with compounds containing phosphorus and sulfur, including DNA. Fang et al. [64] produced silver nanoparticles by reducing AgNPs with tea extract, and the synthesized silver nanoparticles exhibited 96.05% and 61.87% inhibition of *pseudopeptalotiopsis theae* (*P. theae*) and *E. coli*, respectively, indicating their good antibacterial effect. ZOP-NPs-AgNPs have a good antibacterial effect, and the main mechanism of action may be that AgNPs combine with the cell membrane and cell wall, thereby affecting the internal environment of bacteria [65] or damaging bacterial DNA and producing reactive oxygen species, thus killing bacteria. During the formation of AgNPs, some impurities in the polysaccharide were removed, which improved the polysaccharide’s reduction performance [66,67]. The ZOP-NPs-AgNPs prepared by the optimum technology have a higher silver chelating rate and better dispersion, enabling them to better combine with the surface of the bacterial cell membrane, thereby enhancing the antibacterial performance.

### 3.4. Immunomodulatory Activity of ZOP-NPs-AgNPs

Many studies have shown that plant polysaccharides have various biological activities. The immunological and antitumor activities of plant polysaccharides have been widely studied, and polysaccharides have also been proven to be one of the main active components of fungi. However, there are few reports on the immunological activity of plant polysaccharide silver nanoparticles. In this paper, the immunological activity of ZOP-NPs-AgNPs prepared under the optimal process was studied in vitro.

#### 3.4.1. Cell Proliferation Activity

**Figure 6:**
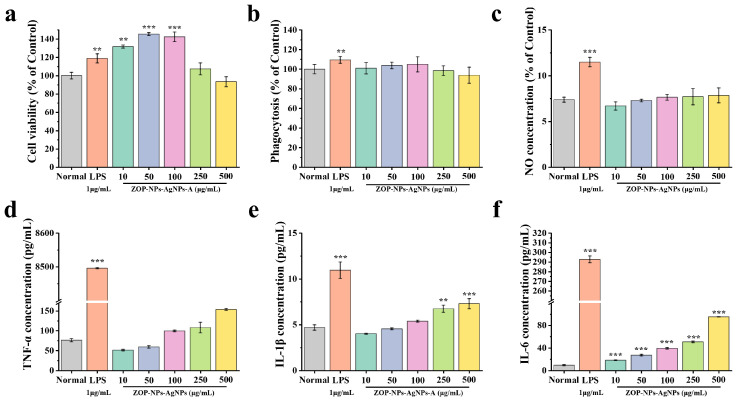
(**a**) Cell proliferation activity. (**b**) Cell phagocytosis. (**c**) Volume of NO produced by cells. (**d**) TNF-α concentration. (**e**) IL-1β concentration. (**f**) IL-6 concentration. Statistical difference against the blank control group (** *p* < 0.01 and *** *p* < 0.001.).

Macrophages can recognize pathogenic substances and remove them directly, which is a key component of the immunomodulatory system [68]. The CCK-8 method was used to study the effect of ZOP-NPs-AgNPs on the survival of RAW 264.7 cells. ZOP-NPs-AgNPs at concentrations of 10–250 μg/mL promoted the proliferation of RAW 264.7 cells without toxicity (Figure 6a). When the concentration of ZOP-NPs-AgNPs was 10–100 μg/mL, the proliferation of RAW 264.7 cells was better than that of the LPS positive control group, and there was a significant difference between ZOP-NPs-AgNPs and the blank control group (*p* < 0.01). When the concentration of ZOP-NPs-AgNPs was 250 and 500 μg/mL, there was no significant difference between the samples and the blank group (*p* > 0.05), and the cytotoxicity was lower when the concentration was 500 μg/mL. Within this concentration range, cell proliferation activity first increased and then decreased with increasing concentration, with the proliferation index reaching 1.45 at 50 μg/mL.

#### 3.4.2. Cell Phagocytic Activity

Studies have shown that macrophage phagocytosis is the first key step in the immune response. Under the induction of LPS, not only were the cell proliferation and phagocytosis abilities enhanced, but also the macrophages could release a large number of cytokines and inflammatory mediators. These mediators can resist the invasion of pathogenic microorganisms, but excessive secretion will cause an inflammatory reaction, so the LPS-induced macrophage activation model is used as an inflammatory model [69]. The neutral red uptake method was used to study the phagocytosis of RAW264.7 cells by ZOP-NPs-AgNPs. The effect of ZOP-NPs-AgNPs on phagocytic activity increased in a concentration-dependent manner in the range of 10–100 μg/mL, but the overall trend was not obvious (Figure 6b). The phagocytosis index decreased at 250 and 500 μg/mL concentrations with increasing concentration, which was lower than the normal level and showed inhibition. Compared with the blank group, there was no significant difference (*p* > 0.05), indicating that ZOP-NPs-AgNPs could not significantly enhance the phagocytic activity of RAW264.7 cells.

#### 3.4.3. The Effect Test of NO Secretion by Macrophages

NO is one of the main reactive oxygen species (ROS) released by macrophages when attacking foreign bodies, which can effectively kill bacteria and play a key role in host defense [70]. In the test concentration range (Figure 6c), it can be seen that ZOP-NPs-AgNPs influenced the ability of macrophages to release NO, but there was no significant difference compared with the blank group (*p* > 0.05). The release of NO increased with increasing concentration and reached the highest level at 500 μg/mL, but there was still a big difference compared with the positive control group. At concentrations of 100–500 μg/mL, ZOP-NPs-AgNPs promoted the secretion of the cytotoxic molecule NO, which played an important role in apoptosis.

#### 3.4.4. The Determination of the Cytokine Secretion Level

RAW264.7 cells are important participants in both nonspecific immunity and specific immune response and play an important role in the immunomodulatory activity [71]. They can initiate the innate immune response by recognizing infectious pathogens, thereby inhibiting the proliferation of various tumor cells and the invasion of microorganisms [72]. Once activated, macrophages can kill pathogens directly by phagocytosis and indirectly by secreting inflammatory mediators such as TNF-α, IL-1β, and IL-6 [73]. ZOP-NPs-AgNPs can promote the secretion of TNF-α, IL-1β, and IL-6 in macrophages at specific concentrations (Figure 6d–f). There was an extremely significant increase in the secretion of IL-1β and IL-6 at concentrations of 250 μg/mL and 500 μg/mL compared to the blank control (*p* < 0.01). The secretion of each cytokine increased in a concentration-dependent manner, and ZOP-NPs-AgNPs inhibited the secretion of IL-1β and TNF-α at low concentrations (10–100 μg/mL). When the concentration of ZOP-NPs-AgNPs was 500 μg/mL, the secretion concentrations of TNF-α, IL-1β, and IL-6 were 153.72, 7.29, and 95.41 pg/mL, respectively. However, the maximum production of cytokines was not higher in any of the three groups than in the LPS-positive control group. These results confirm that ZOP-NPs-AgNPs can promote the secretion of TNF-α, IL-1β, and IL-6 at specific concentrations and that ZOP-NPs-AgNPs have a better immune-enhancing activity at high concentrations.

This study showed that ZOP-NPs-AgNPs, as a kind of metal nanoparticles synthesized by a green pathway, not only possess the physical and chemical properties and antibacterial activity of silver nanoparticles but also acquire some immunomodulatory activities from ZOP. Their structure was characterized by UV-Vis, FT-IR, TEM, SEM, XRD, and TGA. It was found that the silver chelation rate of ZOP-NPs-AgNPs prepared by the optimum process was 92.17%, with a particle size of less than 20 nm, PDI of 0.299, and zeta potential of −32.83 mV. These characteristics are consistent with the physicochemical properties of nano-silver and indicate good thermal stability. In the antibacterial experiment, it was found that ZOP-NPs-AgNPs had certain antibacterial and bactericidal effects on Gram-negative bacteria and Gram-positive bacteria; among them, ZOP-NPs-AgNPs had the best antibacterial and bactericidal effect against *Staphylococcus aureus*, which may be because ginger and nano-silver have certain antibacterial and bactericidal effects. When synthesized into ZOP-NPs-AgNPs through a green method, they achieve a higher silver chelation rate, which enhances their biological activity. Furthermore, ZOP-NPs-AgNPs significantly enhanced the proliferation activity of RAW 264.7 cells and, to some extent, improved their phagocytic activity, thereby activating macrophages. They also demonstrated immunomodulatory capabilities by boosting the secretion of NO, TNF-α, IL-1β, and IL-6.

The present study explores the antibacterial and immunomodulatory activities of ZOP-NPs-AgNPs through in vitro experiments. However, the in vitro model is unable to fully replicate the complex physiological environment found in vivo, where cells are influenced by a multitude of factors, such as the circulatory and immune systems and intercellular interactions. Therefore, in vivo verification is a necessary extension as it can provide comprehensive and biologically relevant information, revealing the mechanisms of action, biodistribution, pharmacokinetic properties, and the safety profile of ZOP-NPs-AgNPs. In vivo experiments can further validate their antibacterial and immunomodulatory effects and explore their interactions with other biomolecules and cells, thereby compensating for the limitations of in vitro studies. Future work will focus on conducting in vivo experiments to provide accurate and comprehensive scientific evidence for the development and application of ZOP-NPs-AgNPs.

## 4. Conclusions

This study successfully optimized the synthesis process of ZOP-NPs-AgNPs using single-factor experiments and response surface methodology, and characterized the resulting nanoparticles. The optimized ZOP-NPs-AgNPs exhibited significantly stronger bacteriostatic and bactericidal efficacy against *Staphylococcus aureus* compared to *Escherichia coli*. Critically, this work demonstrates that ZOP serves as an effective, green, and operationally convenient agent for the preparation of AgNPs. Furthermore, the findings demonstrate that ZOP-NPs-AgNPs possess immunomodulatory activity, as evidenced by their ability to enhance the proliferation and phagocytic activity of RAW 264.7 macrophages and stimulate the secretion of immune mediators (NO, TNF-α, IL-1β, IL-6). While the precise mechanisms underlying this immunomodulatory activity remain to be fully elucidated, the optimized synthesis protocol established here and the demonstrated antibacterial and immunomodulatory properties of ZOP-NPs-AgNPs provide a foundation for further mechanistic studies and exploration of potential applications.

## Figures and Tables

**Table 1:** Independent variables and levels of the experimental design.

Variables	Level		
	−1	0	1
A: V(AgNO_3_):V(polysaccharide)	2.5:1	3:1	3.5:1
B: Extraction temperature (°C)	50	60	70
C: Extraction time (min)	2	3	4

**Table 2:** Box–Behnken experimental design and the results for silver chelation rates.

Run Numbers	V (AgNO_3_): V (Polysaccharide) (A)	Extraction Temperature (B) (°C)	Extraction Time (C) (min)	Chelate Rate (%)
1	−1	−1	0	88.51
2	1	−1	0	89.01
3	−1	1	0	88.97
4	1	1	0	87.83
5	−1	0	0	88.63
6	1	0	0	87.98
7	−1	0	1	88.61
8	1	0	1	88.85
9	0	−1	−1	89.63
10	0	1	−1	90.47
11	0	−1	1	90.52
12	0	1	1	89.79
13	0	0	0	92.79
14	0	0	0	92.92
15	0	0	0	92.33
16	0	0	0	92.25
17	0	0	0	92.25

**Table 3:** Analysis of variance for the experimental results.

Source	Sum of Squares	Df	Mean Square	F-Value	*p*-Value	Significant Level
Model	48.07	9	5.34	63.31	<0.0001	Significant
A	0.53	1	0.53	6.23	0.0413	
B	0.047	1	0.047	0.55	0.4819	
C	0.00045	1	0.0045	0.005334	0.9438	
AB	0.67	1	0.67	7.97	0.0257	
AC	0.89	1	0.89	10.59	0.014	
BC	0.62	1	0.62	7.3	0.0305	
A^2^	29.16	1	29.16	345.63	<0.0001	
B^2^	7.08	1	7.08	83.9	<0.0001	
C^2^	5.18	1	5.18	61.39	<0.0001	
Residual	0.59	7	0.084			
Lack of Fit	0.18	3	0.059	0.57	0.6649	Not significant
Pure Error	0.41	4	0.1			
Correlation Total	48.66	16				

A: V(AgNO_3_):V(polysaccharide). B: Extraction temperature (°C). C: Extraction time (min).

## Data Availability

All data generated or analyzed during this study are available from the corresponding author upon reasonable request.
